# Micronodular PEComa of the appendix: a case report

**DOI:** 10.3389/fmed.2026.1765443

**Published:** 2026-05-22

**Authors:** Li-Jing Jiang, Chao-Qun Wang, Zheng-Guo Xu

**Affiliations:** Department of Pathology, Affiliated Dongyang Hospital of Wenzhou Medical University, Dongyang, Zhejiang, China

**Keywords:** appendix, case report, granular, PEComa, perivascular epithelioid cell tumor

## Abstract

Appendiceal micronodular perivascular epithelioid cell tumor (PEComa) is an extremely rare mesenchymal neoplasm. Historically termed “appendiceal granular cell nodules” or “granular degeneration of smooth muscle,” this entity has recently been reclassified as an indolent variant within the PEComa family, with only isolated cases reported. We report a case of a 23-year-old man with a 4-year history of recurrent right lower quadrant pain, who underwent laparoscopic appendectomy. Histologic examination revealed multiple micronodular nests of epithelioid cells scattered within the muscularis propria, featuring abundant eosinophilic granular cytoplasm and small, round nuclei without significant pleomorphism or mitotic activity. Immunohistochemical analysis showed diffuse positivity for SMA, Desmin, and HMB-45, weak-to-moderate nuclear staining for TFE3, and a Ki-67 proliferation index of less than 1%, supporting the diagnosis of micronodular PEComa of the appendix. This case underscores a rare and frequently underrecognized mesenchymal lesion of the appendix. Further accumulation of cases is necessary to elucidate its pathogenesis, molecular profile, and long-term clinical course.

## 1 Introduction

Perivascular epithelioid cell tumors (PEComas) are rare mesenchymal neoplasms composed of epithelioid-to-spindle cells that typically display a perivascular distribution and co-express melanocytic and myogenic markers (1). Micronodular PEComa of the appendix, first defined by Tran and Fanaian in 2020, represents an exceptionally rare subtype (2). This entity comprises scattered epithelioid nodules within the muscularis propria, with only a few cases documented to date. Due to its subtle histologic features and small size, it can be mistaken for a degenerative or reactive process. This report aims to contribute an additional case to the limited literature and discusses its clinicopathologic characteristics, differential diagnosis, and potential molecular basis.

## 2 Case description

A 23-year-old male patient was admitted to our hospital on 2 August 2025, due to recurrent right lower abdominal pain persisting for over 4 years. The pain was described as persistent and dull in nature, mild in intensity, and not associated with nausea, vomiting, chills, fever, or changes in bowel habits. The patient had no history of tuberous sclerosis complex (TSC). The laboratory findings, including complete blood count and biochemical parameters, revealed no significant abnormalities. Color Doppler ultrasound revealed a tubular echo in the right lower abdomen, suggesting possible appendicitis. Enhanced CT of the lower abdomen showed slight thickening of the appendix without significant wall thickening or high-density shadows within the lumen; the surrounding fat planes were clear. Laparoscopic appendectomy was performed on 4 August 2025.

**FIGURE 1:**
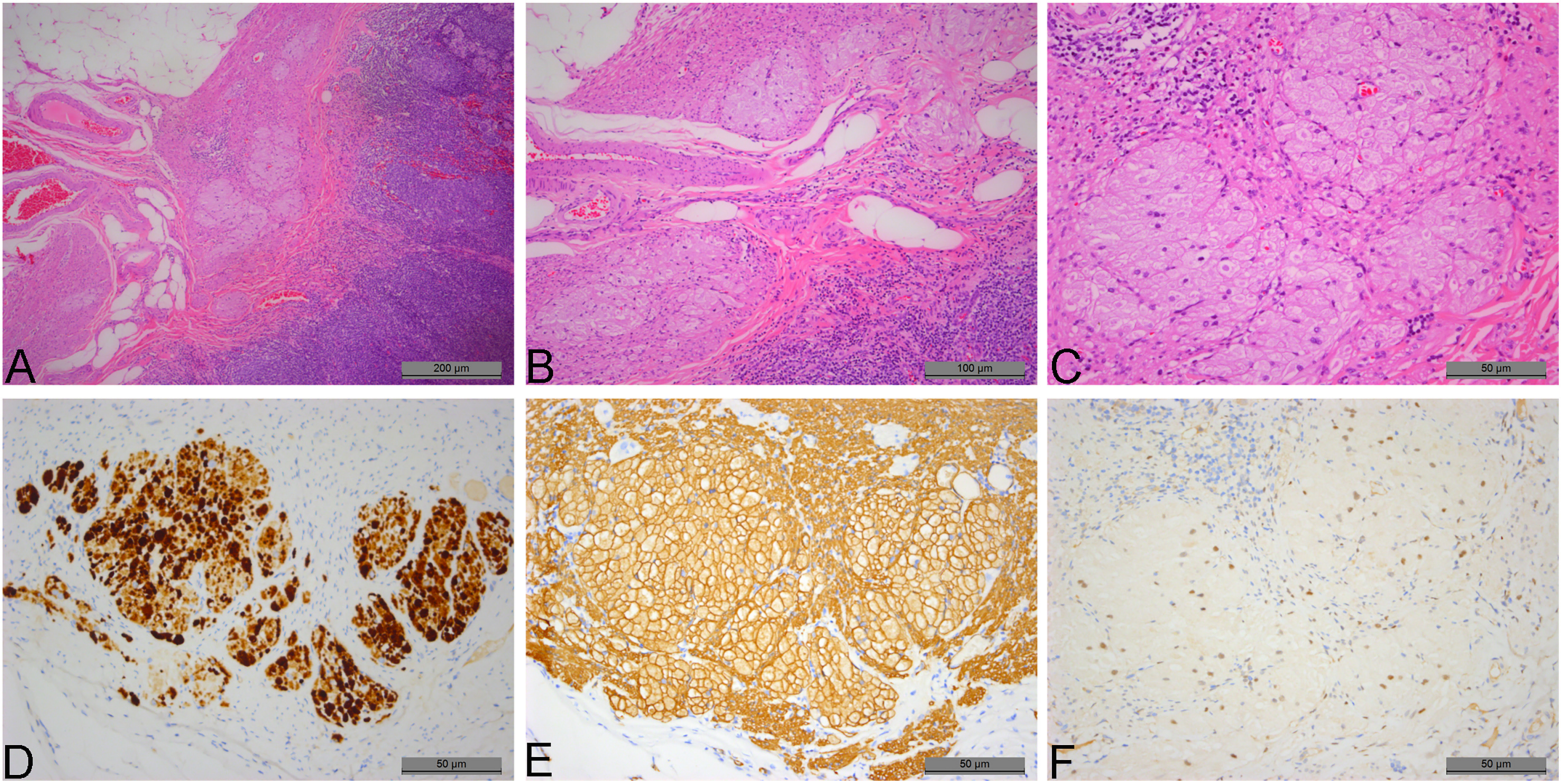
Micronodular perivascular epithelioid cell tumor (PEComa) of the appendix and immunohistochemical staining of tumor cells within epithelioid micronodular nests. **(A)** At low magnification, scattered micronodular nests of epithelioid cells are observed within the muscularis propria (×50). **(B)** At medium magnification, the nests exhibit a micronodular growth pattern (×100). **(C)** At high magnification, the nests are composed of epithelioid cells with abundant granular cytoplasm and small round nuclei, showing no atypia or mitotic activity (×200). **(D)** Tumor cells within the epithelioid micronodular nests demonstrate strong granular cytoplasmic positivity for HMB-45 (×200). **(E)** Tumor cells within the epithelioid micronodular nests show diffuse and strong positivity for SMA (×200). **(F)** Tumor cells within the epithelioid micronodular nests display weak to moderate nuclear positivity for TFE3 (×200).

Pathological gross examination showed an appendix measuring 6 cm in length and 0.6 cm in diameter. The serosal surface was smooth with no obvious fibrinous exudate. Fecal impaction was observed within the lumen, with no evidence of perforation. Microscopic examination revealed well-preserved appendiceal architecture. Fecal impaction was noted at the tip of the appendix, and the lamina propria contained numerous plasma cells. Scattered multiple cellular nests were observed within the muscularis propria. These nests were composed of epithelioid cells with abundant eosinophilic, finely granular cytoplasm and small, round nuclei showing no atypia or mitotic activity. The size of the epithelioid cell nests ranged from 0.1 to 0.4 mm (Figures 1A–C).

Immunohistochemical staining demonstrated that the epithelioid cell nests exhibited diffuse and strong membranous positivity for SMA and Desmin, strong granular cytoplasmic positivity for HMB-45, and weak to moderate nuclear positivity for TFE3 (Figures 1D–F). Stains for CK, Melan-A, S-100, CD68, CgA, Syn, and CD56 were negative. The Ki-67 proliferation index was less than 1%.

Pathological diagnosis: Micronodular PEComa of the appendix.

At the latest follow-up (8 months after surgery), the patient reported marked improvement of abdominal pain. No clinical evidence of recurrence was observed.

## 3 Discussion

Appendiceal micronodular PEComa is an exceptionally rare mesenchymal tumor of the appendix, with only isolated case reports documented to date. This rare histological entity was first described in 1959 by Churg and Work as “appendiceal granular cell nodules” (3). In 1971, Sobel et al. termed it “granular degeneration of smooth muscle” in the appendix, proposing that it represented degenerative smooth muscle cells within the muscularis propria, a view that prevailed for a considerable period (4). In 2020, based on its dual expression of myogenic and melanocytic markers, Tran and Fanaian identified its true nature as a PEComa and first proposed the designation “appendiceal micronodular PEComa” (2). Subsequently, in 2021, Anderson et al. added three further cases, supporting the reclassification of these lesions as genuine PEComas (5).

Perivascular epithelioid cell tumors represent a rare group of mesenchymal tumors. According to the chapter “Tumors of uncertain differentiation” in the 2020 WHO Classification of Tumors of Soft Tissue and Bone (6), the PEComa family includes angiomyolipoma (AML), lymphangioleiomyomatosis (LAM), and PEComa, not otherwise specified (PEComa-NOS). PEComa-NOS encompasses tumors with distinctive morphological and immunohistochemical features. Histologically, they typically exhibit a spindle-to-epithelioid appearance, display characteristic perivascular growth, and possess clear to finely granular eosinophilic cytoplasm. Immunohistochemically, they show co-expression of myogenic and melanocytic markers. PEComa-NOS has a wide anatomical distribution, occurring in sites such as the pancreas, lung, gastrointestinal tract, female genital system, abdomen, pelvis, retroperitoneum, urinary tract, and skin, while primary occurrence in the appendix is exceedingly rare (7). In the present case, the scattered epithelioid cell nests within the appendiceal muscularis propria, along with their histological morphology and immunohistochemical profile, were consistent with a PEComa, leading to the final diagnosis of “appendiceal micronodular PEComa.” Early reports of PEComa family tumors involving the appendix include cases such as the pleomorphic angiomyolipoma described by Prasad et al. (8). However, these lesions presented as discrete mass-forming tumors and differed significantly from the micronodular, incidental lesions described in the current case. It is noteworthy that microscopic examination revealed plasma cell infiltration and lymphoid follicular hyperplasia in the mucosal layer, along with fecal impaction leading to luminal stenosis and obstruction. Therefore, the micronodular PEComa is unlikely to be the direct cause of the patient’s abdominal pain. Instead, the intraluminal fecal impaction and associated mucosal inflammation may better explain the patient’s chronic symptoms.

To date, all reported cases of appendiceal micronodular PEComa have demonstrated benign biological behavior, with no instances of recurrence or metastasis, consistent with an indolent clinical course (2, 5). According to the risk stratification system proposed by Folpe et al. (9), PEComas lacking adverse features such as large tumor size (≥5 cm), infiltrative growth, cytologic atypia, increased mitotic activity, necrosis, and vascular invasion are considered benign. In the present case, the lesion was extremely small (≤0.4 mm), showed no cytologic atypia or mitotic activity, and exhibited no evidence of necrosis or vascular invasion. These findings are consistent with the criteria for benign PEComa and therefore support an indolent biological behavior. However, due to the limited number of cases documented so far, its long-term biological behavior and prognosis remain to be further observed. Surgical resection remains the treatment of choice for most PEComas. Complete excision is generally associated with favorable outcomes, particularly in cases lacking high-risk histologic features. In previously reported cases of appendiceal micronodular PEComa, simple appendectomy appears to be sufficient, with no evidence of recurrence or metastasis to date (2, 5).

In contrast to conventional gastrointestinal PEComas, appendiceal micronodular PEComa does not form a distinct mass. Microscopically, the cell nests are dispersed and are often discovered incidentally in appendectomy specimens. Conventional gastrointestinal PEComas, most commonly found in the colorectum and small intestine, typically present as larger, clinically apparent masses (10). The differential diagnosis also includes granular cell tumor, which usually presents as a well-demarcated, solitary, solid nodule, most frequently located in the submucosa but potentially involving the muscularis propria. It is typically positive for S-100 and negative for HMB-45 on immunohistochemistry (11).

The pathogenesis of PEComas primarily involves two mutually exclusive mechanisms. The first is loss-of-function mutations in TSC1 or TSC2, which occur in the majority of TFE3 rearrangement negative cases and are frequently accompanied by TP53 alterations. These mutations, whether germline in TSC or sporadic, lead to constitutive activation of the mTOR pathway, thereby providing a rationale for targeted therapy with mTOR inhibitors (12–14). The second mechanism involves TFE3 gene rearrangements, which define a distinct molecular subgroup that is mutually exclusive with TSC1/TSC2 mutations. PEComas harboring TFE3 rearrangements characteristically exhibit diffuse and strong nuclear TFE3 immunoreactivity (13, 15, 16); however, weak-to-moderate or focal staining has occasionally been reported in fusion-positive cases (17, 18), underscoring that immunohistochemistry alone cannot entirely rule out this alteration.

In the present case, the patient had no clinical history of TSC, and immunohistochemistry demonstrated only weak-to-moderate nuclear TFE3 expression. Consequently, neither the clinical history nor the immunophenotype was sufficient to confidently assign the tumor to either the TSC/mTOR-mutant or the TFE3-rearranged molecular subgroup. This diagnostic ambiguity represents a significant limitation of our study, as it directly stems from the lack of molecular characterization. In the current molecular era, precise classification of PEComas is critical; it not only refines prognostication but also has immediate therapeutic implications, as mTOR inhibitors are preferentially considered for tumors with TSC1/TSC2 loss. Although RNA-based fusion assays (e.g., Archer FusionPlex Sarcoma or Pan-Solid Tumor Panel) represent the gold standard for detecting TFE3 rearrangements and identifying novel gene fusions, such analyses were not performed in the present case owing to refusal by the patient and family. Furthermore, this absence of molecular data precludes evaluation of other recently described genomic alterations in PEComas, such as ATRX mutations (19). Therefore, future studies incorporating comprehensive molecular profiling, including next-generation sequencing and fusion detection, will be essential to elucidate the oncogenic drivers and to determine whether the molecular landscape of appendiceal micronodular PEComa aligns with that of conventional PEComas at other sites.

## 4 Conclusion

Appendiceal micronodular PEComa is an exceedingly rare mesenchymal tumor of the appendix. This case report enhances the understanding of rare mesenchymal tumors of the appendix, aiding in the prevention of misdiagnosis or underdiagnosis. Due to the scarcity of reported cases worldwide, its pathogenesis, molecular profile, and long-term prognosis require further investigation and validation. Future efforts should focus on accumulating more cases to verify its biological behavior, explore potential molecular abnormalities, and establish standardized diagnostic and management guidelines.

## Data Availability

The raw data supporting the conclusions of this article will be made available by the authors, without undue reservation.
